# Effect of COVID-19 vaccination appointment letters on uptake by sociodemographic characteristics: a regression discontinuity analysis in Sweden, December 2020 to September 2021

**DOI:** 10.1093/eurpub/ckaf097

**Published:** 2025-06-23

**Authors:** Georgios Varotsis, Ulf Hammar, Carl Bonander, Per Lundmark, Beatrice Kennedy, Maria F Gomez, Mats Martinell, Oliver J Dyar, Anna Sarkadi, Robert Kristiansson, Helena Svaleryd, Tove Fall

**Affiliations:** Molecular Epidemiology, Department of Medical Sciences, Uppsala University, Uppsala, Sweden; Molecular Epidemiology, Department of Medical Sciences, Uppsala University, Uppsala, Sweden; School of Public Health and Community Medicine, University of Gothenburg, Gothenburg, Sweden; Molecular Epidemiology, Department of Medical Sciences, Uppsala University, Uppsala, Sweden; Molecular Epidemiology, Department of Medical Sciences, Uppsala University, Uppsala, Sweden; Diabetic Complications Unit, Department of Clinical Sciences in Malmö, Lund University Diabetes Centre, Malmö, Sweden; Department of Public Health and Caring Sciences, Uppsala University, Uppsala, Sweden; Child Health and Parenting (CHAP), Department of Public Health and Caring Sciences, Uppsala University, Uppsala, Sweden; Child Health and Parenting (CHAP), Department of Public Health and Caring Sciences, Uppsala University, Uppsala, Sweden; Department of Public Health and Caring Sciences, Uppsala University, Uppsala, Sweden; Department of Economics, Uppsala University, Uppsala, Sweden; Molecular Epidemiology, Department of Medical Sciences, Uppsala University, Uppsala, Sweden

## Abstract

Ensuring high vaccination coverage is vital, particularly during a pandemic. While pre-booked appointment letters have shown promise in vaccination campaigns, their effectiveness in specific sociodemographic groups remains to be explored. Our study evaluated the effect of pre-booked appointment letters on COVID-19 vaccine uptake within different sociodemographic groups using a quasi-experimental methodology. In Uppsala County, Sweden, residents born between 1962 and 1971 received pre-booked COVID-19 vaccination letters starting 24 May 2021, while younger residents received SMS prompts for self-booking starting 7 June 2021. Through a regression discontinuity design, we used the intervention cut-off at birth year 1971 to assess the effectiveness of the letters to increase vaccine uptake compared to the SMS campaign. Our analysis included 96 194 individuals born between 1962 and 1981, examining vaccination within 90 days post-eligibility as primary outcome. We investigated effects within sociodemographic groups, assessed household spillover effects, and performed negative control analyses using neighbouring counties. Adults just above the cut-off had an odds ratio of 1.3 (95% CI 1.10–1.53) of being vaccinated than those just below, with a 1.97 percentage point increase (95% CI: 0.45–3.50) from a baseline of 91.95%. The intervention showed effectiveness within most sociodemographic strata. No effects were found in negative control counties, nor were there household spillover effects. Pre-booked appointment letters are effective at boosting vaccination uptake, even in diverse sociodemographic groups. While our findings come from COVID-19 vaccination, they align with evidence from various immunization programs, suggesting that personalized communications can achieve equitable vaccine coverage across different healthcare settings.

## Introduction

Vaccines are pivotal in reducing human suffering and death [1–3] attributed to life-threatening infectious diseases. Immunizations represent cost-effective public health investments linked to favourable economic and social impacts for individuals and the broader community [2]. However, achieving universal vaccine uptake requires strategic approaches, especially among disadvantaged groups.

In recent years, health experts have emphasized the need for broad health interventions across the entire population, tailored to specific needs and levels of disadvantage [4]. This approach is particularly relevant given the disproportionate impact of several infectious diseases on specific sociodemoghraphics [5], as evidenced by the higher rates of infection and severe disease during the COVID-19 pandemic among these populations [6–8].

At the same time, universal vaccine interventions rely on successful campaigns, especially among disadvantaged groups [9]. Studies have observed lower vaccine uptake among younger adults, men, and those with low income, foreign-born status, and low educational attainment, especially for COVID-19 [5, 10–12]. Older age is generally associated with higher vaccine uptake, highlighting the need for targeted strategies to address vaccine hesitancy also in younger populations [13, 14]. Public trust in authorities and science seem to influence vaccine acceptance [15, 16]. However, despite efforts to tackle distrust, uptake may remain lower among disadvantaged groups due to vaccine hesitancy driven by accessibility challenges, personal beliefs, government distrust, and variations in health literacy [9, 17].

To address low vaccine uptake and high hesitancy, nudges—subtle and low-cost behavioural interventions[18]—have gained momentum [19]. Mailing pre-booked appointment letters has shown promise as a vaccination nudge in Uppsala County, Sweden, where studies have demonstrated efficacy among teenagers [20] and military conscripts [21]. While a randomized clinical trial (RCT) in Italy demonstrated the effectiveness of this intervention in adults aged 50–59 [22], its effectiveness on specific sociodemographic groups with lower COVID-19 vaccination rates and higher vaccine hesitancy, as noted in the literature [10, 11, 17], remains to be explored.

Our study aimed to address this gap by using a regression discontinuity (RD) design to evaluate the effect of mailing pre-booked vaccination appointment letters on COVID-19 vaccine uptake in adults. We hypothesized that providing pre-booked appointments through personalized mailed letters could enhance vaccine uptake by eliminating obstacles to accessing vaccination information and self-scheduling appointments, including an examination of intervention effects within different sociodemographic groups. We further explored the partner spillover effect within households to examine any indirect intervention effect on partners. The quasi-experimental RD design methodology was chosen given its suitability for estimating causal effects when there is a clear cut-off point [23, 24] in observational data.

## Methods

### Study design

The age-based policy in Uppsala County (total adult population as of 31 December 2020: 309 685), Sweden, with its distinct cut-off point (year of birth 1971), served as the foundation for our quasi-experimental design. In early 2021, as COVID-19 vaccines became available in Sweden, counties were directed by national health authorities to prioritize vaccination in a specified order. After completing phases 1–3 (detailed in Supplementary Material A.1), phase 4 targeted the general population aged 18–59 years, using a stepwise approach beginning with the oldest individuals. Counties had autonomy to define age intervals, set timelines, and choose communication methods for inviting eligible individuals. The decision to implement pre-booked appointment letters for those born in 1971 and earlier, and not in those born later, balanced multiple factors by the local health authorities: age-related risk of severe disease, anticipated vaccination interest, cancellation risks, and logistical constraints. While the pre-booked appointment letters were less flexible, this approach aimed to ensure higher coverage. The birth year 1971 threshold thus served as a pragmatic cut-off point, approximating age 50, to balance vaccine prioritization with available resources. While this cut-off could have been set at adjacent years, it probably provided a convenient round number for administrative purposes.

With RD design, individuals on either side of the threshold are compared, assuming those just above and below the cut-off are similar, except for their exposure to the intervention (having received a vaccination letter or not). The Uppsala County Council (Region Uppsala) sent Swedish-language letters (approximately 260 words) containing pre-booked vaccination appointments, comprehensive timing and location details, and explicit rescheduling instructions to all residents born between 1962 and 1971. Recipients could attend or actively cancel without consequences, maintaining future rebooking rights. In contrast, those born after 1971 received brief SMS messages (approximately 24 words) in Swedish, directing them to self-book appointments through their 1177 account at a vaccination centre of their choice, either online or by phone. All vaccines were provided free of charge, and a facsimile of the vaccination letter appears in Supplementary Fig. S1, and the SMS message in Supplementary Fig. S2.

### Data sources and potential effect modifiers

This study used pseudonymized individual-level data from Swedish national health and demographic registers (2020–2021). Vaccination data came from the National Vaccination Register. Population and individual data on county of residence, household partners, sex at birth, education attained, duration of residency in Sweden, and country of birth were obtained for the year 2020 from the Total Population Register (RTB), while individual annual disposable income expressed in hundreds of Swedish Krona (SEK) for 2020 was derived from The Longitudinal Integrated Database for Health Insurance and Labour Market Studies (LISA) Register, both maintained by Statistics Sweden (SCB). Information about medications consistent with being in a medical-risk group and hence, was based on the National Patient Register and the National Prescribed Drug Register, both held by the National Board of Health and Welfare.

We defined our primary outcome as receiving the first dose of any COVID-19 vaccine within 90 days after the vaccination opening date. The opening dates in 2021 were categorized based on the year of birth in accordance to the county’s vaccination schedule program: vaccination slots were made available on May 24 for those born between 1962 and 1971, on May 31 for those born between 1972 and 1976, and on June 7 for individuals born between 1977 and 1981. Additionally, individuals born between 1962 and 2003 with a medical condition predisposing them to develop severe COVID-19 were offered vaccination earlier, starting on May 3. However, we did not distinguish these individuals from the general population as it was uncertain from the data who received priority due to their medical condition. We also aimed to investigate the potential household spillover effect of vaccination letters on a partner’s vaccine uptake. We examined partnered individuals born between 1972 and 1981 living with partners born between 1962 and 1981on 31 December 2020. The outcome was defined in the same way as the primary analysis, but the intervention was defined as having a partner born between 1962 and 1971.

Concerning potential effect modifiers, individuals were classified according to institutional confidence of their country of birth into three categories; born in Sweden, born abroad in a high-trust country, and born abroad in a low-trust country based on a revised Hofstede-Inglehart trust index [25]. A variable reflecting the duration of residence in Sweden in years until 31 December 2020 was derived for all individuals (born in Sweden, short: born abroad with 10 years or less residence, long: born abroad with more than 10 years residence). The highest education level attained was classified into three categories: primary school, upper secondary school, and university. Annual disposable income was categorized into three groups (low, middle, and high) using tertiles. The classification was based on the distribution of disposable income within the individual’s corresponding sex and birth year in 2021. The age of each individual was estimated on 1 June 2021 while the term ‘sex’ pertains to the biological categorization of women and men. Finally, a classification into the high- and low-risk groups was made based on medical conditions as defined by the SWECOV registry (Supplementary Material A.2).

As part of the sensitivity analysis, we performed a negative control analysis using data separately for Gävleborg and Stockholm, two neighbouring counties to Uppsala. For details regarding the communication strategies, vaccination opening dates, and approaches of these two counties, see Supplementary Material A.3.

The project has been approved by the Swedish Ethical Review Authority (DNR 2022-01355-02).

### Statistical analysis

In the primary and spillover analyses, we performed kernel-weighted logistic regression with vaccination within 90 days (yes/no) as outcome and letter intervention (yes/no) as exposure using Epanechnikov kernel with Imbens and Kalyanaraman bandwidth [26]. For the primary analysis, the age of the individual was included in the model, while for the spillover analysis, the age of the spouse was included. Age was modelled using linear splines with a single knot at 49.5 years (the age marking the commencement of the vaccination letter intervention for individuals born in 1971) to estimate odds ratios around the cut-off point.

Subgroup analyses were investigated independently for each sociodemographic characteristic (education attainment, sex, trust level of country of birth, duration of residence, and medical-risk group status) by adding interaction terms between intervention and the specific moderator, as well as the main effect of that moderator, to the main model. Each characteristic was analysed separately, and we did not test the significance of the interaction term itself, focusing on within-group effects rather than between-group comparisons.

In the secondary analysis, we employed local polynomial method (rdrobust R library). We maintained consistency with the primary analysis by employing the same kernel and bandwidth methods, but with stratification by the same candidate moderator categories.

The identifying assumption in the empirical model is that the treatment assignment changes discontinuously at the cut-off. Since pre-booked appointment letters were sent only to individuals born in 1971 or earlier, and because age cannot be manipulated, this requirement is satisfied. Nonetheless, we perform several validation tests as part of the sensitivity analysis. First, to ensure that the results are not driven by differences between populations just above and below the cut-off, we examine whether vaccination letter recipients near the cut-off are similar to control individuals in terms of observable characteristics. Second, we vary the bandwidth using another econometrics method with higher variance but lower bias, outlined in Calonico *et al*. [27]. Third, to further assess the robustness of our findings, we conduct a negative control analysis separately for Gävleborg and Stockholm counties, followed by comparative analyses of the ORs between the Uppsala–Gävleborg and Uppsala–Stockholm pairs.

All *P*-values and the 95% CIs presented are based on regular (non-clustered) robust (sandwich) standard errors (see Supplementary Material A.4).

Analyses were performed using R version 4.3.2 with appropriate packages for RD analysis (details in Supplementary Material A.5).

## Results

### Study population

Our study population included all 96 194 individuals who were registered as living in Uppsala County on 31 December 2020, born between 1962 and 1981. A total of 47 917 individuals were born between 1962 and 1971 and thus received the intervention and 48 277 were born between 1972 and 1981 and did not receive the intervention (Table 1). The younger group had higher educational attainment (52.9% versus 42.2% with university education), a lower proportion of individuals classified with a high-risk status (24.0% versus 37.8%), and a smaller percentage who received vaccination ahead of schedule (25.3% versus 36.6%). The proportion of individuals vaccinated born in 1972 was 91.95%. For details on the baseline characteristics, please see Table 1.

**Table 1.** Baseline characteristics

	Group with appointment letter (*N* = 47 917)	Group with self-booking (*N* = 48 277)
Sex		
Women	23 716 (49.5%)	23 850 (49.4%)
Men	24 201 (50.5%)	24 427 (50.6%)
Age (mean in years)^a^	54.4 (2.8)	44.5 (2.9)
Trust level of country of birth^b^		
Born in Sweden	37 611 (78.5%)	34 755 (72.0%)
High-trust	6073 (12.7%)	7276 (15.1%)
Low-trust	4230 (8.8%)	6233 (12.9%)
Missing	3 (<0.1%)	13 (<0.1%)
Duration of residence		
Born in Sweden	37 611 (78.5%)	34 755 (72.0%)
Long (over 10 years)	8095 (16.9%)	8265 (17.1%)
Short (10 years or less)	2184 (4.6%)	5249 (10.9%)
Missing	27 (0.1%)	8 (<0.1%)
Education attained		
University	20 209 (42.2%)	25 554 (52.9%)
Upper secondary school	22 338 (46.6%)	17 357 (36.0%)
Primary school	4911 (10.2%)	4455 (9.2%)
Missing	459 (1.0%)	911 (1.9%)
Medical-risk group^c^		
High	18 135 (37.8%)	11 592 (24.0%)
Low	29 782 (62.2%)	36 685 (76.0%)
Vaccinated ahead of schedule^d^		
Yes	17 543 (36.6%)	12 207 (25.3%)
No	30 374 (63.4%)	36 070 (74.7%)
Vaccinated within 90d^d^		
Yes	43 934 (91.7%)	41 823 (86.6%)
No	3983 (8.3%)	6454 (13.4%)
Vaccination period		
Earliest date	27 December 2020	30 December 2020
Latest date	21 August 2021	4 September 2021
Annual disposable income (median, in hundreds of SEK)		
High income	3593 (3130–4140)	3640 (3202–4154)
Middle income	1627 (1479–1808)	1684 (1510–1877)
Low income	331 (28–679)	274 (37–624)

Data are in *n* (%), median (IQR), mean (SD).

a Age calculated as (2021 − Age_year) + (6 − Age_month)/12.

b Based on the revised Hofstede–Inglehart trust index.

c Based on the list of medical conditions curated by SWECOV (Supplementary material A.2).

d Counting from the opening date corresponding to the year of birth.

### Primary analysis

**Figure 1.**
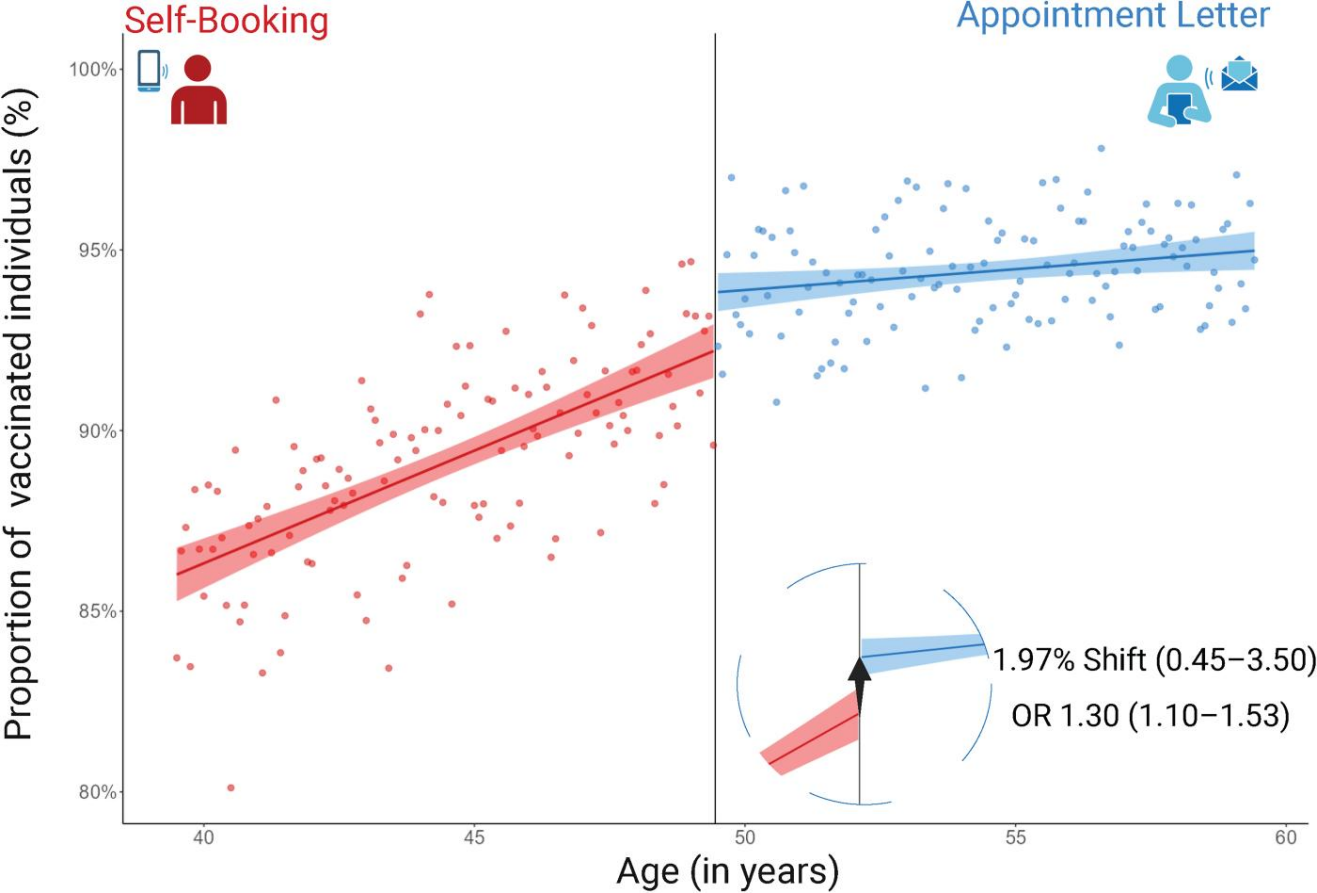
Regression discontinuity plot with a linear regression slope superimposed on both sides of the cut-off line illustrating the proportion of individuals (%) vaccinated within 90 days post-eligibility, with regression lines with 95% confidence intervals. Each point on the plot represents the proportion of vaccinated individuals within a 1-month age range. The intervention group (Appointment Letter) appears on the right of the reference vertical line (black line at 49.5 years, representing approximate age of individuals born in the 1971 cut-off year), while the comparison group (Self-Booking) is displayed on the left of the reference line. The inner circle zooms in on the cut-off, displaying the percentage change in vaccinations with a 95% confidence interval, along with the odds ratio (OR) for appointment letters recipients compared to those self-scheduling.

**Figure 3.**
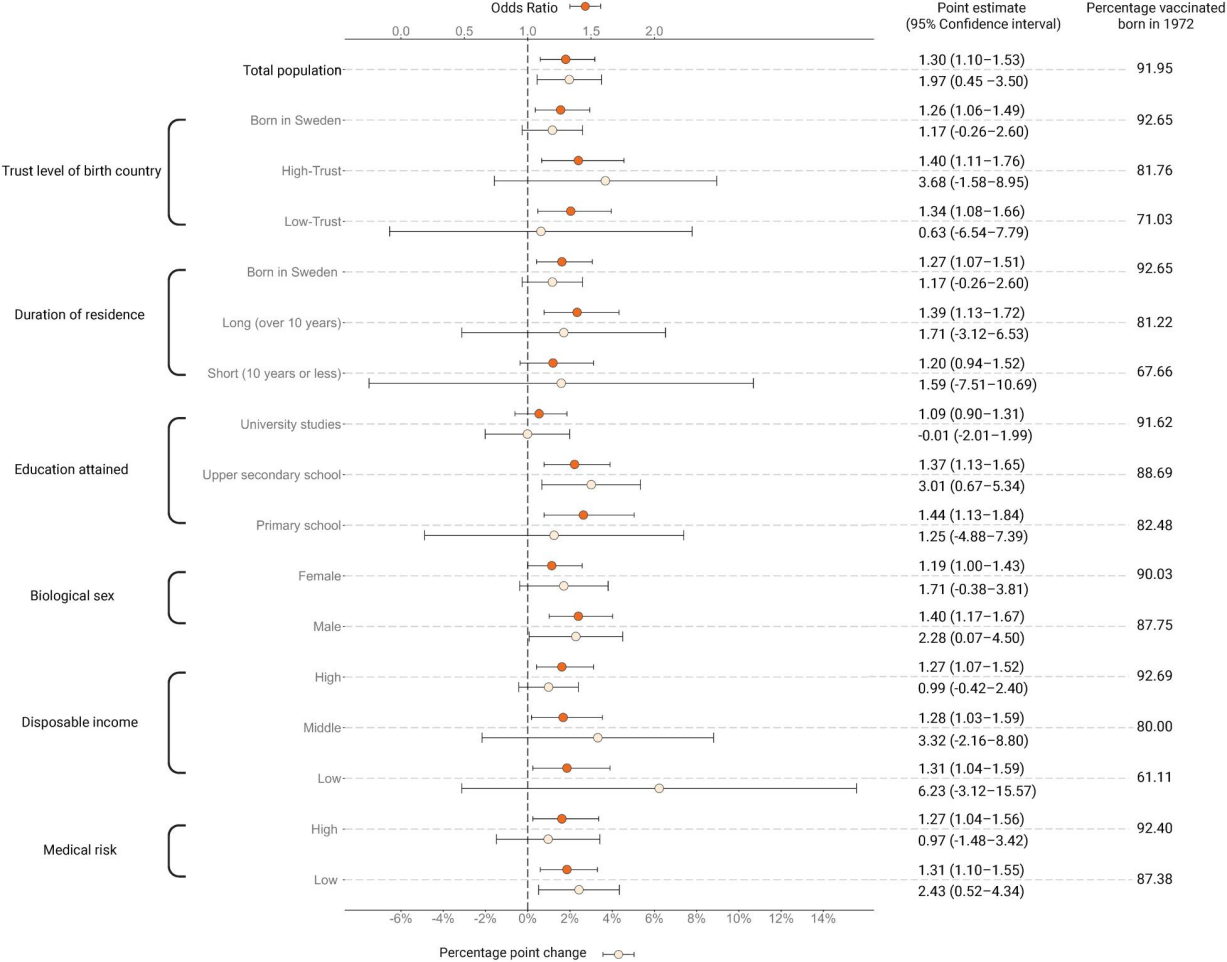
Estimated intervention effect on the total population and within sociodemographic groups based on the optimal bandwidths. Data are odds ratios (95% confidence interval) and percentage point change (95% confidence interval) for the estimated effect of vaccination appointment letters for total population and different sub-groups, and percentage of individuals vaccinated among those born in 1972, just above the birth year cut-off. The comparison group is individuals who could only self-book vaccination appointments.

The RD plot for the overall population supports a linear functional form between vaccine uptake and age in the full age span included in the study, with a discontinuity in vaccine uptake at the policy threshold (Fig. 1). Using the optimal bandwidth of 5.39 (Imbens and Kalyanaraman method) we observed an OR of 1.30 (95% CI: 1.10–1.53) of being vaccinated within 90 days from the opening among those who received the intervention compared to self-booking around the cut-off point (Fig. 3).

In the subgroup analysis (Fig. 2), we examined the intervention effect separately within each sociodemographic category. The intervention showed effectiveness in individuals with primary school education (OR 1.44, 95% CI: 1.13–1.84) and those with upper secondary education (1.37, 95% CI: 1.13–1.65), as well as within income categories [(low 1.31 (95% CI: 1.04–1.59), middle 1.28 (95% CI: 1.03–1.59), high 1.27 (95% CI: 1.07–1.52)] and in men (1.40, 95% CI: 1.17–1.67). In separate analyses by country of birth using the revised Hofstede–Inglehart trust index [25], we found evidence of an effect within those born in high- (1.40, 95% CI: 1.11–1.76) and low-trust countries (1.34, 95% CI: 1.08–1.66) as well as those born in Sweden (1.26, 95% CI: 1.06–1.49) or who had been residing in Sweden for over a decade (1.39, 95% CI: 1.13–1.72). For individuals residing in Sweden for less than a decade, we found no evidence of an effect, possibly due to a lack of power (1.20, 95% CI: 0.94–1.52). We also stratified the analysis on the estimated risk of severe COVID-19 based on medical data, and found evidence of an effect of the intervention in both high- (1.27, 95% CI: 1.04–1.56) and low-risk individuals (1.31, 95% CI: 1.10–1.55) (Fig. 3).

**Figure:**
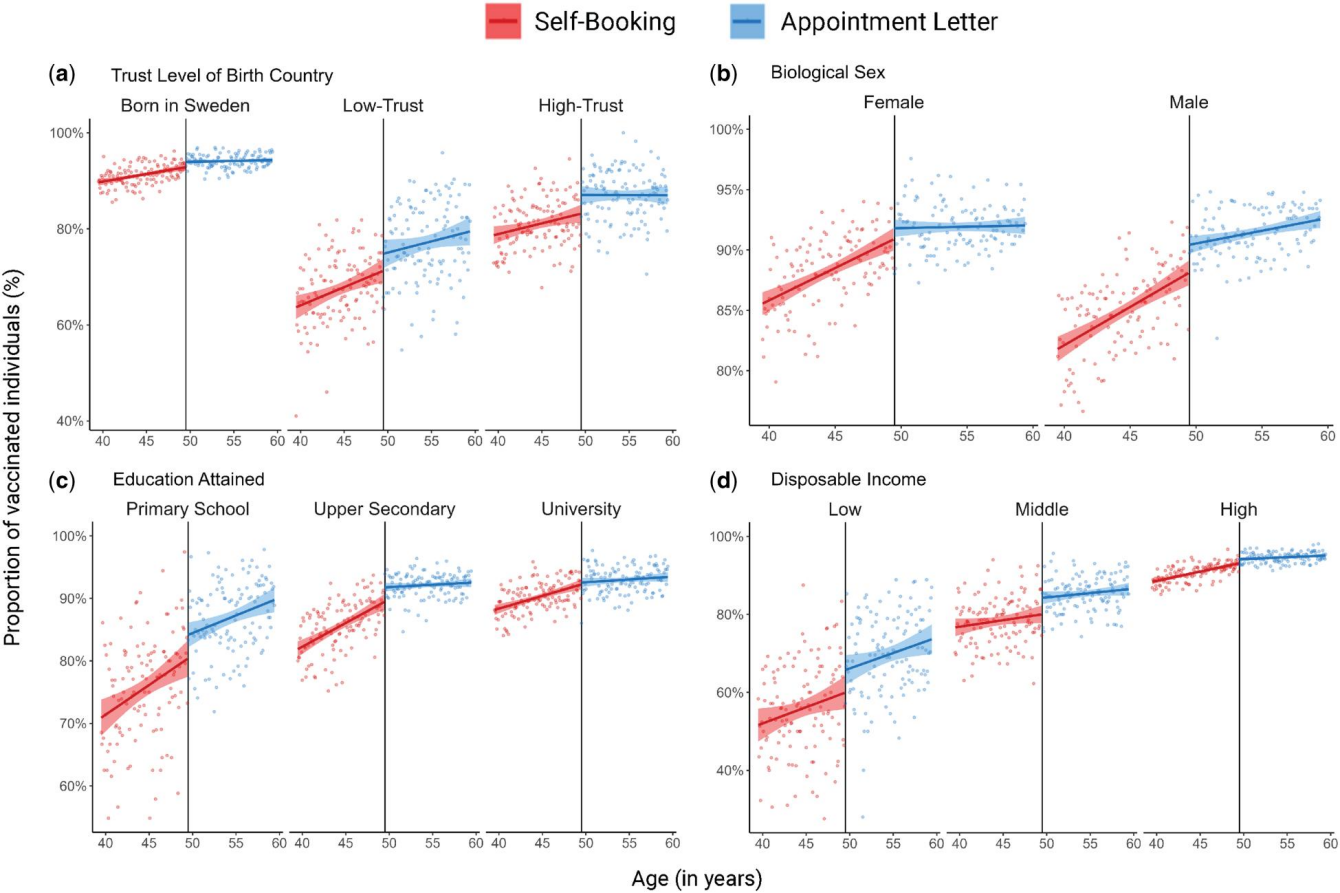
**Figure 2**. Stratification of the regression discontinuity plot with a linear regression slope superimposed on both sides of the cut-off by potential moderators. The plots show the proportion of individuals (%) vaccinated within 90 days post-eligibility, with regression lines with 95% confidence intervals. Each point on the plot represents the proportion of vaccinated individuals within a 1-month age range. The intervention group (Appointment Letter) appears on the right of the reference vertical line (black line at 49.5 years, representing approximate age of individuals born in the 1971 cut-off year), while the comparison group (Self-Booking) is displayed on the left of the reference line. The moderators shown include (a) trust level of country of birth, (b) sex, (c) education attained, and (d) annual disposable income. Please note that distinct *y*-axis scales have been utilized for each panel to facilitate the comprehension of the changes in vaccination levels within different moderators.

### Secondary analysis

The secondary analysis (Fig. 3) was used to estimate the effect of the intervention using the local polynomial method, a method with higher variance, but potentially lower bias. Using the optimal bandwidth, we observed a 1.97 (95% CI: 0.45–3.50) percentage point increase in vaccinations from a baseline (among people born in 1972) of 91.95%. The secondary analysis supported an intervention effect among those attending upper secondary school (3.01, 95% CI: 0.67–5.34, baseline: 88.69%), men (2.28, 95% CI: 0.07–4.50, baseline: 87.75%), and those with low medical risk of severe COVID-19 (2.43, 95% CI: 0.52–4.34, baseline: 87.38%). Detailed results from the primary and secondary analyses can be found in Fig. 3 and Supplementary Table S1.

### Spillover analysis

In the spillover analysis, we restricted our data to those 26 522 individuals born between 1972 and 1981 who were partnered with someone born between 1962 and 1981. Only 20.1% (*N* = 5587) had a partner targeted by the intervention with the vast majority being women (81.8%, *N* = 4572). We did not find any evidence of a spillover effect between partners as indicated in the main spillover model (OR 0.98; 95% CI: 0.71–1.34), possibly due to a lack of power.

### Sensitivity analysis

In the sensitivity analysis, no discernible discontinuities were observed around the threshold in education attainment, income, the number of individuals vaccinated ahead of schedule, or any other background variables (Supplementary Table S2), which supports that our results are specific to the intervention. We also repeated the primary analysis using a narrower bandwidth (2.72) as instructed by Calonico *et al.* [27], to assess the sensitivity of the results to the intervention responses of individuals closer to the year of birth cut-off. The point estimate was very similar, but as expected the CI widened as the number of observations was reduced (1.27, 95% CI: 0.99–1.61). In addition, in a separate analysis, we focused on a subset of the data comprising the 66 444 individuals who had remained unvaccinated on the day of the official opening of vaccination appointments and found a slightly larger OR of 1.34 (95% CI: 1.15–1.57) with a markedly higher percentage point shift of 3.50 (95% CI: 1.51–5.49). In the negative control analysis, the ORs did not indicate any difference at the 1971 birth year cut-off in Gävleborg (0.97, 95% CI: 0.86–1.09) or Stockholm (1.01, 95% CI: 0.96–1.06). These estimates were significantly lower than the ones in Uppsala (*P*-values = .005 and .0008, respectively). However, the non-parametric linear analysis showed a difference at the cut-off in Stockholm: 0.95 (95% CI: 0.27–1.64). The discontinuity plots for Gävleborg and Stockholm can be found in Supplementary Fig. S3. More detailed results from the sensitivity analysis are shown in Supplementary Table S3.

## Discussion

Our study employed a RD design to examine the influence of mailing pre-booked COVID-19 vaccination appointment letters on adult vaccine uptake within diverse sociodemographic groups.

### Key findings

We found results consistent with a direct policy effect at the birth year 1971, with adults just above the intervention cut-off having 30% higher odds of receiving vaccination compared to those just below, with a 1.97 percentage point rise in vaccine uptake from a baseline of 91.95%. These results align with previous interventions: an Italian RCT reported a 3.2 percentage point increase for pre-set appointments [22], and a US-based experimental study found default scheduling boosted vaccination intentions by 6 percentage points, starting from a baseline of 70% [28]. Monetary incentives in Sweden led to a 4.2 percentage point increase from a 71.6% baseline [29].

Our primary analysis indicated that the intervention increased vaccination odds across most sociodemographic groups, including those with lower education levels, men, and individuals born in Sweden and abroad. The non-linear analysis confirmed these effects among those with upper secondary education, men, and individuals with low medical risk. These results align with previous studies assessing the same intervention in Uppsala County with different analytical methodologies, targeting specific demographics like teenagers [20] and male military conscripts [21]. In addition, our results are consistent with previous research on influenza vaccination initiatives in the Netherlands, where invitation letters sent based on year of birth to those aged 65 or older seemed most effective among men, those with lower education levels, and those in medical-risk groups [30].

We found no household spillover effect between partners, perhaps due to limited sample size. Nonetheless, the result aligns with a Dutch influenza program, where a favourable spillover effect could not be discerned [30].

### Methodological considerations

Unlike studies measuring COVID-19 and influenza vaccination intentions, which can be inaccurate proxies for vaccination rates, our study used objective vaccination data. Also, our comparison group received SMS prompting self-booking, suggesting our effects might be conservative compared to no intervention. As shown by a US study, SMS alone increased vaccination rates by 3.6 percentage points [19]. Sensitivity analyses excluding individuals vaccinated before the intervention (due to high medical risk or other priority status) led to larger effect estimates, with no evidence of discontinuity in pre-intervention vaccination rates.

Our intervention combined two components: the medium of communication (letter versus SMS) and the message content (appointment versus reminder), both potentially contributing to its effectiveness with its impact differing when examining different sociodemographic groups separately. Traditional letters may be more effective than SMS due to their perceived formality and legitimacy, while pre-scheduled appointments could reduce procrastination compared to reminders.

The analysis of the 90-day post-availability interval occurred during a period of ongoing vaccine safety monitoring. While some vaccine safety concerns were raised in mid-2021, they had limited direct relevance to our study population (aged 40–59) during the letter intervention period (after 24 May 2021) (see Supplementary Material A.12).

Furthermore, while our study examined COVID-19 vaccination in Sweden, the effectiveness of personalized communications aligns with evidence from various vaccination programs. For instance, personalized vaccine communication interventions have shown significant success, with one study demonstrating a 61% higher likelihood of vaccination and sustained improvements in vaccine confidence [31]. Text message reminders in the US [32] and electronic letters for patients with diabetes in Denmark [33] increased influenza vaccination. These suggest a broader applicability of simple and scalable communication strategies for improving immunization coverage across different contexts.

Finally, pre-intervention vaccination rates influence intervention effectiveness [19, 29], with higher baseline rates typically yielding smaller effects. In our study, the high pre-intervention rates and observed plateau above 90% suggest limited room for improvement, as remaining unvaccinated individuals likely had strong vaccine hesitancy or contraindications.

### Strengths and limitations

Our study has several strengths. First, the age-based policy cut-off enables comparison of similar individuals around the threshold, while our register-based data encompassing the entire population aged 40–59 eliminates sampling and response bias [34], particularly valuable for assessing effects in disadvantaged groups less likely to participate in surveys. Our results also remained robust to alternative specifications, bandwidths, and falsification tests, with negative control analyses showing no similar effects in neighbouring counties, except for a likely spurious discontinuity on the absolute scale in Stockholm.

Some limitations warrant consideration. While the RD design offers strong internal validity, unobservable factors around the cut-off point could confound our estimates despite no discontinuity in observable variables. Earlier roll-outs in neighbouring counties could indirectly influence Uppsala's population through information sharing and social networks, potentially increasing vaccination rates in the control group and thus underestimating the true effect of the letter intervention. Additionally, our findings may not be directly transferable to other contexts, such as routine or childhood immunizations, or to individuals outside the studied age group, as RD estimates are based on behaviours observed around individuals born near the birth year 1971. Finally, while our findings come from COVID-19 vaccination, the fundamental principles of personalized communication likely extend beyond pandemic response. Future research should examine how to optimize these communication strategies across different vaccines and public health scenarios to maximize their impact on routine immunization programs.

In conclusion, our study supports pre-booked appointment letters as a scalable intervention that extends beyond COVID-19 vaccination. Their effectiveness among sociodemographic groups, even across those with lower vaccination rates, suggests these personalized communications could enhance uptake for both routine immunizations and future pandemic responses. This approach offers a practical strategy for achieving equitable vaccination coverage across different healthcare settings and immunization programs.

## Supplementary Material

ckaf097_Supplementary_Data

## Data Availability

The data presented in this article contain sensitive personal information and are subject to access restrictions, requiring approval from the Swedish Ethical Review Authority (In Swedish: ‘Etikprovningsmyndigheten’). All scripts containing the code used to generate the variables and conduct analyses are openly accessible through the following link: https://github.com/MolEpicUU/vax-letters-rdd-sweden. Key points Pre-booked vaccination appointment letters increased COVID-19 vaccine uptake compared to an invitation by SMS to self-book online, demonstrating effectiveness in several sociodemographic groups including low education and low-income strata. Our results suggest that personalized invitations can help achieve more equitable vaccine coverage compared to self-booking systems during a pandemic. Appointment letters have the potential to improve uptake of other preventive health services.
