# Supplementary material for: Effect of COVID-19 vaccination appointment letters on uptake by sociodemographic characteristics: a regression discontinuity analysis in Sweden, December 2020 to September 2021
Source: Eur J Public Health. 2025 Jun 23;35(4):795–802. doi: 10.1093/eurpub/ckaf097 (PMC12311344; doi:10.1093/eurpub/ckaf097)
Supplement: ckaf097_Supplementary_Data [file ckaf097_supplementary_data.docx]

Supplementary Material

Table of Contents

[**A.1 Vaccination prioritization** defined by The Public Health Agency (Folkhälsomyndigheten). 1](#_Toc185338547)

[**A.2 Medical conditions** included in the definition of the variable ‘Medical Risk Group’ 2](#_Toc185338548)

[**A.3** **Communication strategies** in neighbouring counties 2](#_Toc185338549)

[**A.4 Adjustment of p-values** for heteroskedasticity robust standard errors 3](#_Toc185338550)

[**A.5 R packages** used in the analyses 3](#_Toc185338551)

[**A.6 *Suppl. Table 1****:* Results from the primary and secondary analyses. 4](#_Toc185338552)

[**A.7 Suppl. Table 2**: Results from the sensitivity analyses investigating discontinuity of background variables. 5](#_Toc185338553)

[**A.8 Suppl. Table 3**: Results from the sensitivity analyses on the employment of narrower bandwidth, the exclusion of previously vaccinated individuals, and the negative control. 5](#_Toc185338554)

[**A.9 Suppl. Figure 1**: Original vaccination letter – Facsimile letter and translation in English 6](#_Toc185338555)

[**A.10 Suppl. Figure 2**: Original SMS message with translation in English 7](#_Toc185338556)

[**A.11 Suppl. Figure 3**: Negative control analyses **-** Regression discontinuity plots on neighboring counties 8](#_Toc185338557)

[**A.12 Vaccine safety concerns** during our study period 9](#_Toc185338558)

[**References** 10](#_Toc185338559)

## **A.1 Vaccination prioritization** defined by The Public Health Agency (Folkhälsomyndigheten).

The national vaccination strategy proceeded in four sequential phases:

1. Phase 1: Healthcare workers, elderly care residents and staff
2. Phase 2: Individuals aged ≥65 years, organ transplant recipients, dialysis patients and their households, and those with certain disabilities
3. Phase 3: Ages 60-64 years and ages 18-59 with underlying conditions associated with an increased risk of severe COVID-19, as outlined in a prespecified list. These conditions included chronic cardiovascular disease (e.g., stroke and hypertension), chronic lung disease (e.g., COPD or severe, unstable asthma), conditions leading to reduced lung function or secretion stagnation (e.g., extreme obesity, neuromuscular diseases, or multiple disabilities), chronic liver or kidney failure, type 1 or type 2 diabetes, immunosuppression due to illness or treatment, and Down syndrome.
4. Phase 4: Remaining general population aged 18-59 years, , starting with the oldest and progressing to younger age groups

Counties were directed by national health authorities to follow this prioritization order but had flexibility in implementing phase 4, including defining specific age intervals and choosing communication methods for vaccine invitations. Source: Nationell plan för vaccination mot covid-19.^1^

## **A.2 Medical conditions** included in the definition of the variable ‘Medical Risk Group’

A binary variable was curated by Olof Östergren (SWECOV) to identify individuals with pre-existing medical condition based on a list maintained by Swedish authorities, including diagnoses, treatments, and medication purchases up to five years prior to or during the index year (2021). This approach ensures inclusion of individuals who may perceive themselves as being at higher risk due to certain conditions, rather than exclusively considering conditions linked to elevated infection risks or severe outcomes. The conditions included cancer, diabetes, dementia, psychological disorders, alcohol and/or drug abuse, diseases of the circulatory system, chronic respiratory diseases, chronic liver disease, kidney disease, neuromuscular disorders, obesity, rheumatoid arthritis, pregnancy, immunocompromised, adrenal insufficiency, and having received organ transplants.

## **A.3** **Communication strategies** in neighbouring counties

In Stockholm County, during phase 4 of the national vaccination strategy, individuals were encouraged to book vaccination appointments through the *1177* hotline or the regional digital platform *Alltid Öppet*, which allowed users to select a preferred vaccination center and time. Individuals without healthcare registration were instructed to schedule appointments through the 1177 healthcare hotline. Communication efforts included digital campaigns on platforms like Facebook and Twitter, as well as traditional media such as television. In Gävleborg County, individuals were encouraged to use either 1177 online platform for booking, especially those with a social security number, or the 1177 hotline for those without healthcare registration.

For Stockholm and Gävleborg, the opening dates in 2021 were also grouped based on the study participants’ year of birth and the local vaccination schedule policies: 1962 to 1966 on May 3; 1967 to 1971 on May 10; 1972 and 1975 on May 17 (Stockholm) and May 24 (Gävleborg); and 1976 and 1981 on May 31.

## **A.4 Adjustment of p-values** for heteroskedasticity robust standard errors

p-values for the main and subgroup analyses and interactions involving variables with two categories (income, sex, comorbidities) were adjusted for heteroskedasticity robust standard errors using the *lmtest* and *sandwich* R libraries. For variables with more than two categories (trust level of birth country, duration of residence and education), the linearHypothesis function (*car* R library) was used instead. In the sensitivity analysis, interaction p-values for the non-parametric method were computed manually. This involved estimating the mean beta values across different categories for each variable of interest and subsequently using a chi-square (χ^2^) distribution with 1 and 2 degrees of freedom for two groups and three groups, respectively.

## **A.5 R packages** used in the analyses

The library ggplot2 (v. 3.4.3) was used to generate the plots, rddapp (v. 1.3.2) to estimate bandwidth using the Imbens-Kalyanaraman method, survey (v. 4.2-1) to apply kernel weights to the logistic regression, car (v. 3.1-2) for linear hypothesis testing of the effect modifiers, sandwich (v. 3.0-2) for robust variance-covariance estimation, lmtest (v. 0.9-40) to perform Wald tests, rdrobust (v. 2.1.1) to estimate the bandwidth based on Calonico et al.^2^ to perform the non-parametric linear analysis.

## **A.6 *Suppl. Table 1****:* Results from the primary and secondary analyses.

| **Model** | **Logistic (OR)** | **p-value (robust)** | **Non-parametric linear (percentage points)** | **p-value (robust)** |
| --- | --- | --- | --- | --- |
| **Main intervention effect (Uppsala)** | 1·30 (1·10–1·53) | 0·0016 | 1·97 (0·45–3·50) | 0·011 |
| **Interactions** |  |  |  |  |
| **Trust level of birth country** |  |  |  |  |
| Born in Sweden | 1·26 (1·06–1·49) | 0·0089 | 1·17 (-0·26–2·60) | 0·11 |
| High-Trust | 1·40 (1·11–1·76) | 0·0041 | 3·68 (-1·58–8·95) | 0·17 |
| Low-Trust | 1·34 (1·08–1·66) | 0·0083 | 0·63 (-6·54–7·79) | 0·86 |
| **Duration of residence** |  |  |  |  |
| Born in Sweden | 1·27 (1·07–1·51) | 0·0062 | 1·17 (-0·26–2·60) | 0·11 |
| Long (over 10 years) | 1·39 (1·13–1·72) | 0·0021 | 1·71 (-3·12–6·53) | 0·49 |
| Short (10 years or less) | 1·20 (0·94–1·52) | 0·14 | 1·59 (-7·51–10·69) | 0·73 |
| **Education attained** |  |  |  |  |
| University studies | 1·09 (0·90–1·31) | 0·37 | -0·01 (-2·01–1·99) | 0·99 |
| Upper secondary school | 1·37 (1·13–1·65) | 0·0012 | 3·01 (0·67–5·34) | 0·012 |
| Primary school | 1·44 (1·13–1·84) | 0·0029 | 1·25 (-4·88–7·39) | 0·69 |
| **Biological sex** |  |  |  |  |
| Female | 1·19 (1·00–1·43) | 0·054 | 1·71 (-0·38–3·81) | 0·11 |
| Male | 1·40 (1·17–1·67) | 0·0002 | 2·28 (0·07–4·50) | 0·044 |
| **Disposable income** |  |  |  |  |
| High | 1·27 (1·07–1·52) | 0·0070 | 0·99 (-0·42–2·40) | 0·1698 |
| Middle | 1·28 (1·03–1·59) | 0·0029 | 3·32 (-2·16–8·80) | 0·24 |
| Low | 1·31 (1·04–1·65) | 0·023 | 6·23 (-3·12–15·57) | 0·19 |
| **Medical risk** |  |  |  |  |
| High | 1·27 (1·04–1·56) | 0·018 | 0·97 (-1·48–3·42) | 0·44 |
| Low | 1·31 (1·10–1·55) | 0·0019 | 2·43 (0·52–4·34) | 0·013 |

## **A.7 Suppl. Table 2**: Results from the sensitivity analyses investigating discontinuity of background variables.

| **Background variable** | **OR (95% CI)** | **p-value (robust)** |  |
| --- | --- | --- | --- |
| High-trust level of birth country | 0·92 (0·80–1·16) | 0.22 |  |
| Low-trust level of birth country | 1·02 (0·89–1·17) | 0.80 |  |
| Long duration of residence | 1·00 (0·92–1·10) | 0.92 |  |
| Short duration of residence | 0·98 (0·83–1·16) | 0.85 |  |
| University education | 0·95 (0·86–1·05) | 0.31 |  |
| Primary school education | 0·90 (0·77–1·05) | 0.19 |  |
| Males | 1·05 (0·96–1·16) | 0.31 |  |
| High disposable income | 1·09 (0·97–1·23) | 0.16 |  |
| Low disposable income | 0·89 (0·72–1·10) | 0.28 |  |
| High medical risk | 1·00 (0·91–1·11) | 0.95 |  |
| Vaccinated ahead of schedule | 0·92 (0·84–1·01) | 0.10 |  |
| Data are odds ratios (95% robust confidence interval adjusted for robust standard errors), percentage point change (95% confidence interval adjusted for robust standard errors) and p-values (adjusted for robust standard errors and shown with two significant figures and capped at 4 decimals. | | |  |
|  |  |  |  |
|  |  |  |  |
|  |  |  |  |

## **A.8 Suppl. Table 3**: Results from the sensitivity analyses on the employment of narrower bandwidth, the exclusion of previously vaccinated individuals, and the negative control.

| **Model** | **Logistic (OR)** | **p-value (robust)** | **Non-parametric linear (percentage points)** | **p-value (robust)** |
| --- | --- | --- | --- | --- |
| **Main intervention effect (Uppsala)** | 1·30 (1·10–1·53) | 0·0016 | 1·97 (0·45–3·50) | 0·011 |
| With narrower bandwidth (Calonico et. al) | 1·27 (0·99–1·61) | 0·055 | 0·63 (-1·67–2·94) | 0·59 |
| After exclusion of previously vaccinated individuals | 1·34 (1·15–1·57) | 0·0002 | 3·50 (1·51–5·49) | 0·0006 |
| **Intervention effect (Gävleborg)** | 0·97 (0·86–1·09) | 0·48 | -0·51 (-2·13–1·11) | 0·40 |
| **Intervention effect (Stockholm)** | 1·01 (0·97–1·06) | 0·62 | 0·95 (0·27–1·64) | 0·0065 |

## **A.9 Suppl. Figure 1**: Original vaccination letter – Facsimile letter and translation in English

*Suppl. Figure 1:* Example of the original vaccination letter (a) distributed to all residents of Uppsala County in 2021 who were born between 1962 and 1971. The letter featured personalized information including appointment date, time, and vaccination center location for each recipient. The authors have provided a translated version (b) to make the letter’s content accessible to the international audience.


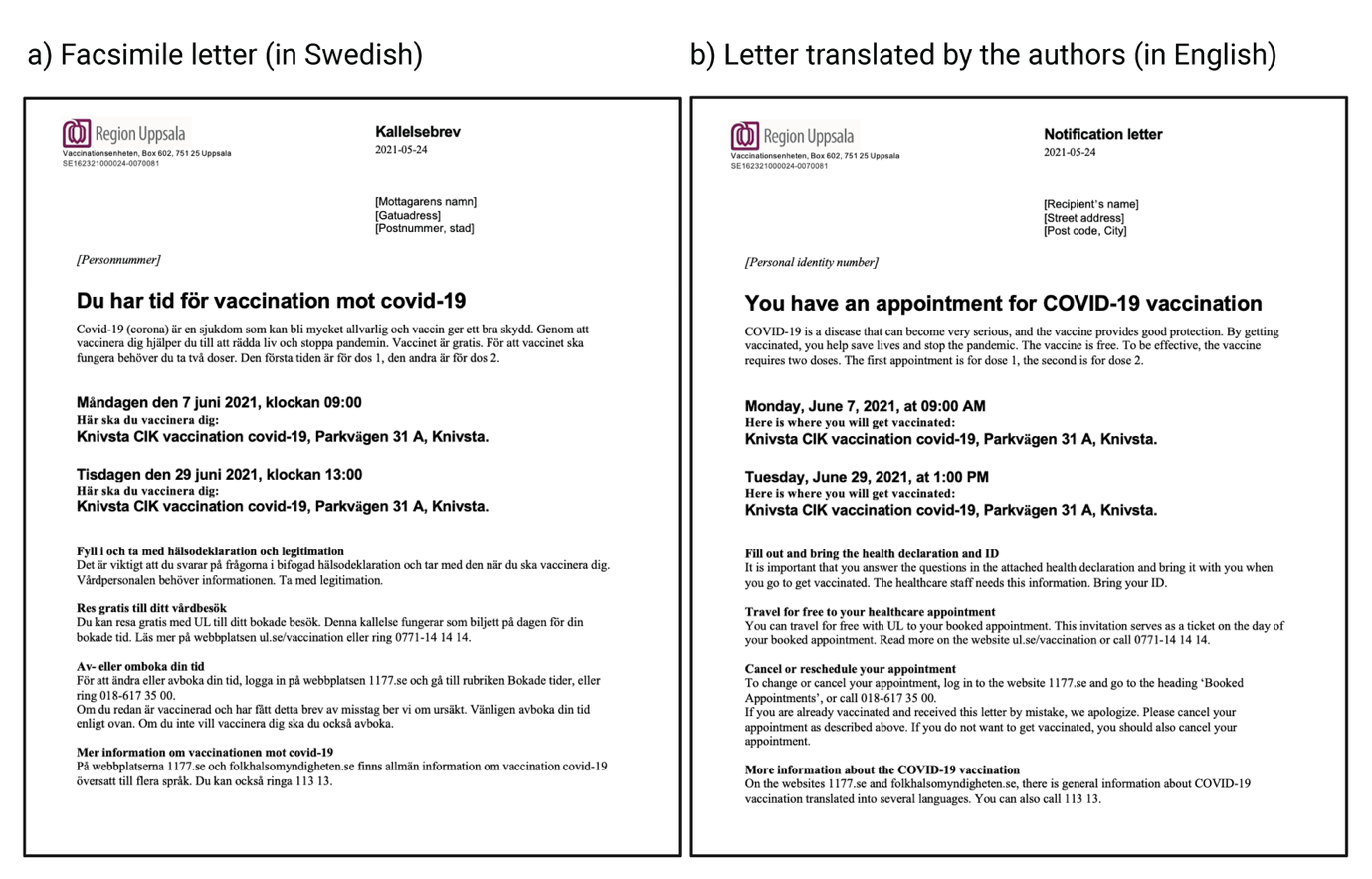


## **A.10 Suppl. Figure 2**: Original SMS message with translation in English

*Suppl. Figure 2:* The original SMS message (a) distributed to all residents of Uppsala County in 2021 who were born after 1971. The message instructed recipients to book their vaccination appointments through their 1177 account. The authors have provided a translated version (b) to make the message’s content accessible to the international readers.


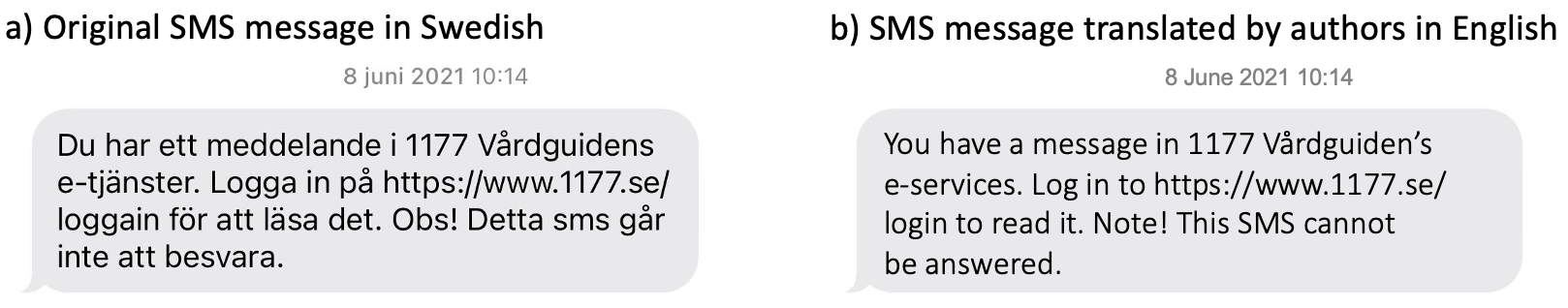


## **A.11 Suppl. Figure 3**: Negative control analyses **-** Regression discontinuity plots on neighboring counties

*Suppl. Figure 3:* Regression discontinuity plot (linear representation) illustrating the proportion of individuals (%) vaccinated within 90 days post-eligibility, with regression lines with 95% confidence intervals for a) Gävleborg and b) Stockholm Counties. Each point on the plot represents the proportion of vaccinated individuals within a one-month age range. The negative control intervention group (“Appointment Letter”) is represented in blue, while the comparison group (“Self-Booking”) is depicted in red. displaying the percentage change in vaccinations with a 95% confidence interval, along with the odds ratio comparing appointment letter recipients to those self-scheduling.


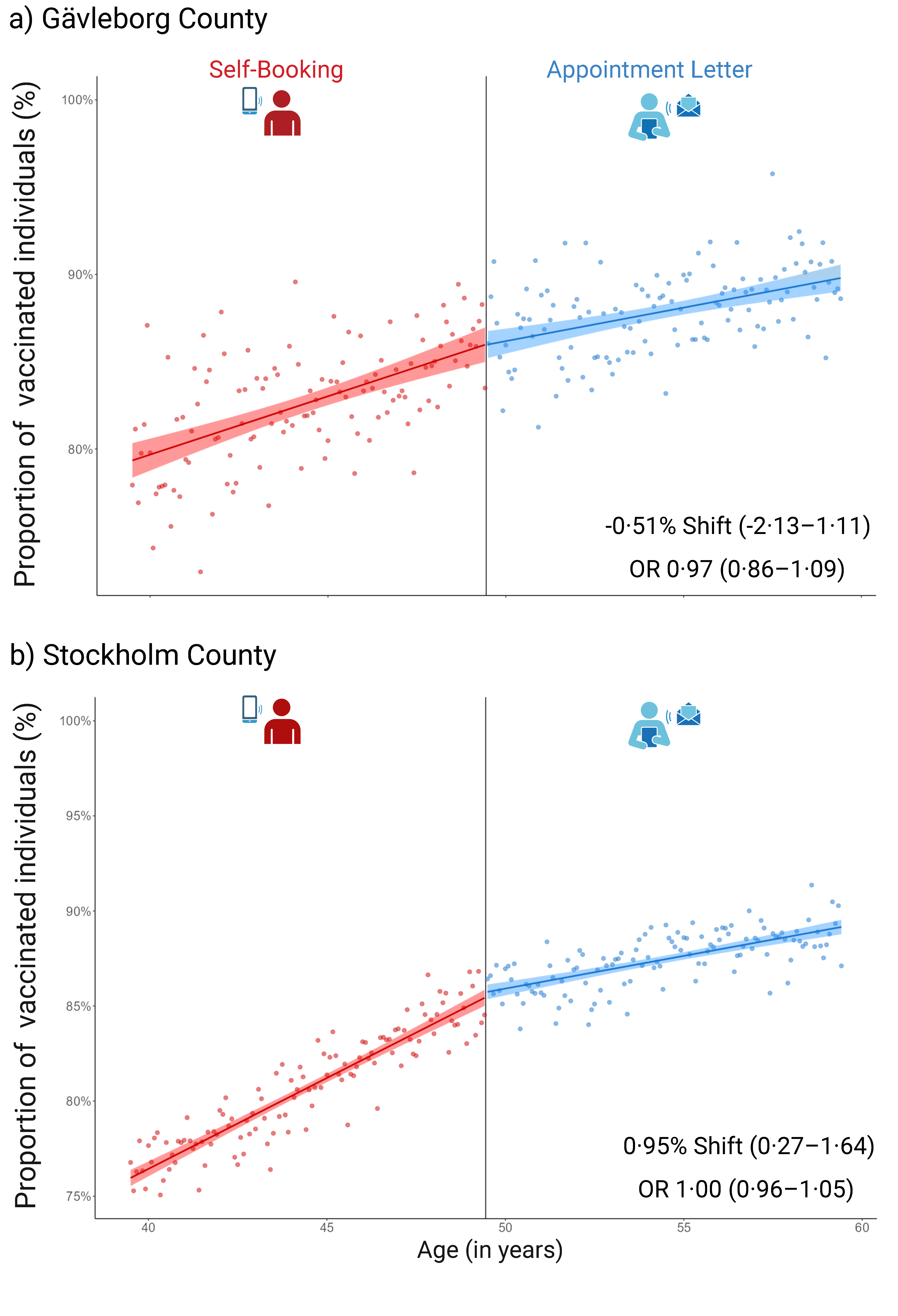


## **A.12 Vaccine safety concerns** during our study period

During March-April 2021, several events related to the AstraZeneca vaccine occurred in Sweden: the Public Health Agency (FoHM) temporarily suspended its use due to concerns over adverse events related to blood clots, while later the vaccine was restricted to only those over 65 years.^3^ Additionally, reports of myocarditis associated with Moderna's vaccine emerged in June 2021,^4^ leading to Sweden later suspending its use for younger adults (under 30) in October 2021.^5^

However, these safety concerns had limited direct relevance to our study population (aged 40-59) during the letter intervention period (after May 24, 2021) for several reasons: 1) The AstraZeneca vaccine was not offered to our age groups during the intervention period, 2) The Moderna-related concerns primarily affected younger age groups, and 3) No major vaccine safety events were reported during our intervention period for vaccines offered to our target age groups.

While earlier media coverage of these safety concerns may have influenced general attitudes towards vaccines, it is unlikely to have differentially affected individuals around our study's age cutoff point (birth year 1971) during the intervention period, supporting the validity of our estimated intervention effects.

## **References**

1 Folkhälsomyndigheten. Nationell plan för vaccination mot covid-19. 2021. https://www.folkhalsomyndigheten.se/contentassets/43a1e203f7344a399367b816e2c7144c/nationell-plan-vaccination-covid-19-delrapport-3.pdf (accessed Dec 10, 2024).

2 Calonico S, Cattaneo MD, Titiunik R. Robust Nonparametric Confidence Intervals for Regression-Discontinuity Designs. *Econometrica* 2014; **82**: 2295–326.

3 Grönt ljus för Astra Zenecas vaccin för 65 år och äldre | Vårdfokus. 2021. https://www.vardfokus.se/nyheter/gront-ljus-for-astra-zenecas-vaccin-for-65-ar-och-aldre/ (accessed Dec 11, 2024).

4 Myokarditkomplikation ovanlig efter sars-cov-2-mRNA-vaccination. https://lakartidningen.se/klinik-och-vetenskap-1/nya-ron/2023/02/myokarditkomplikation-ovanlig-efter-sars-cov-2-mrna-vaccination/ (accessed Dec 11, 2024).

5 Coronavaccinet Nuvaxovid stoppas för personer under 30 år | Kungälvs-Posten. https://www.kungalvsposten.se/nyheter/sverige/coronavaccinet-nuvaxovid-stoppas-for-personer-under-30-ar.f3fae2e0-38a8-4512-9215-98ce8752948a (accessed Dec 11, 2024).
